# Disparities in the access and provision of mental health services as part of primary health care: a case study of Ga-South district in the Greater Accra region

**DOI:** 10.3389/fpubh.2025.1537955

**Published:** 2025-05-09

**Authors:** Abena Boahemaa Adusei, Roberta Naa Barkey Ayiku, Kezia Akosua Naa Amerley Amarteyfio, Eugene Paa Kofi Bondzie, Nhyira Yaw Adjei-Banuah, Abdul-Basit Abdul-Samed, Tolib Mirzoev, Irene Akua Agyepong

**Affiliations:** ^1^Ghana College of Physicians and Surgeons, Accra, Ghana; ^2^London School of Hygiene and Tropical Medicine, London, United Kingdom

**Keywords:** mental health disparity, healthcare inequality, primary health care, health accessibility, qualitative study, mental health service

## Abstract

Mental illness can be as debilitating as physical ailments, yet many mental health patients lack access to quality mental health care in low- and middle-income countries. This study aimed to identify and characterize disparities in access to mental health care, as well as challenges in service provision, in the Ga-South district of Ghana. A qualitative case study design was used, employing purposive and snowball sampling to recruit participants. Sixteen in-depth interviews were conducted with 17 participants, which included one interview with 2 participants. The participants were mental health patients, caregivers of mental health patients, mental health advocates, health promotion officers, and psychiatric nurses in the Ga-South district of Ghana. The findings reveal that stigma, inadequate support, deficiencies in diagnosis and referral, and high treatment costs create substantial disparities in mental health care access. Stigma around mental health constrains both the provision of mental health care by nurses and healthcare-seeking efforts by patients. Diagnosis and referrals of mental health cases are sometimes deficient in the district, with some medical officers and midwives diagnosing mental health conditions inadequately before referrals to psychiatrists. Nurses on the wards also struggle to manage mental health cases, even after receiving mental health training from their facilities. Findings also reveal that some healthcare professionals sometimes exhibit apathy toward mental health issues. The cost of services and medications for patients is also high which prevents patients from seeking care. Additionally, a critical shortage of psychiatrists results in inadequate patient follow-up. The study underscores the urgent need for comprehensive reforms in mental health care delivery to promote inclusion and address disparity issues. It is necessary to ensure equitable access to quality mental health services by addressing workforce shortages and financial barriers, as well as enhancing awareness, training, and efforts to reduce stigma. Prioritizing these reforms will help to create a healthcare system that effectively supports mental health, promoting healthier communities and improving overall health outcomes.

## 1 Introduction

Mental health conditions are defined as clinically significant disturbances in an an individual's cognition, emotional regulation, or behavior, that causes distress or impairment in critical areas of functioning (1). They include anxiety disorders, depression, bipolar disorder, post-traumatic stress disorder, eating disorders, schizophrenia, conduct disorders, and neurodevelopmental disorders (1). The Center for Disease Control (CDC) categorizes and describes mental health disparities as comprising: (1) disparities between the attention given to mental health and that given to other public health issues of comparable magnitude, (2) disparities between the health of persons with mental illness as compared with that of those without, or (3) disparities between populations with respect to mental health and the quality, accessibility, and outcomes of mental health care (2). Before the COVID-19 pandemic, the World Health Organization (WHO) estimated that about 116 million individuals in the African Region suffered from mental health disorders, alongside alarming increases in suicide rates and alcohol abuse among adolescents as young as 13 years (3).

Despite this high burden, mental health care in Africa is underprioritized, with fewer than two mental health professionals per 100,000 people and services mainly concentrated in urban centers, leaving rural and primary care populations severely underserved (3). Less than 11% of African member states offer pharmaceutical and/or psychological therapies at the primary care level, although two-thirds of them have written standards for integrating mental health into primary health care (3). A major barrier is the inadequate funding allocated to mental health compared to other health conditions (3). These mental health disparities are complex challenges mostly influenced by a variety of factors at the individual, community, programme, system, and policy levels (2).

In Ghana, over 2.3 million individuals are affected by mental health issues (4). Patients admitted to mental hospitals in Ghana are categorized into the following three diagnostic groups: Mental and behavioral diseases caused by substance use (42%), schizophrenia (34%), and mood disorders (16%) (33). Mental healthcare delivery faces significant challenges: there are no cohesive mental health care plans, monitoring of the few professionals is ineffective, and there is a severe shortage of psychotropic drugs and competent clinical psychologists (5). According to the World Health Organization (6), only 39 psychiatrists (0.13 per 100,000 persons) serve a population where an estimated 2.3 million require treatment.

Mental health had a ring-fenced budget of 1.4% of the total governmental health expenditure and that puts a strain on an already overburdened system (7). Various governments have made efforts since 1980 to legislate the provision of mental services in Ghana and that brought about the promulgation of the Mental Health Act. However, these legislations have not consistently protected the rights and interests of the mentally ill (7). Mental health patients and their caregivers face stigma and discrimination in their bid to seek health care which usually has an enormous negative impact on the patients and their families.

While previous studies have documented challenges in mental healthcare delivery in Ghana, limited research has focused on the disparities in access to mental health services at the primary health care level. This study aimed to explore and characterize these disparities in the Ga-South district, providing a critical foundation for informing improved policy and practice. It had two specific research objectives; 1) to gain an in-depth understanding of unequal treatment patients face in accessing primary mental health care at the community and facility level 2) to understand the challenges health service providers face in delivering primary mental health care.

## 2 Materials and methods

### 2.1 Study design

This study was designed as a qualitative case study and is part of the larger STOP-NCD programme under the UK National Institute of Health and Care Research (NIHR) Global Health Research Center for Non-Communicable Disease Control in West Africa. The primary aim is to obtain a thick and contextually rich understanding of the disparities in mental health care access at the primary health care level in the Ga-South district of Ghana. Using in-depth interviews, the study explored the experiences and perceptions of mental health patients, caregivers, and service providers, thereby informing the development of targeted interventions to address these disparities.

### 2.2 Study setting

**Figure 1:**
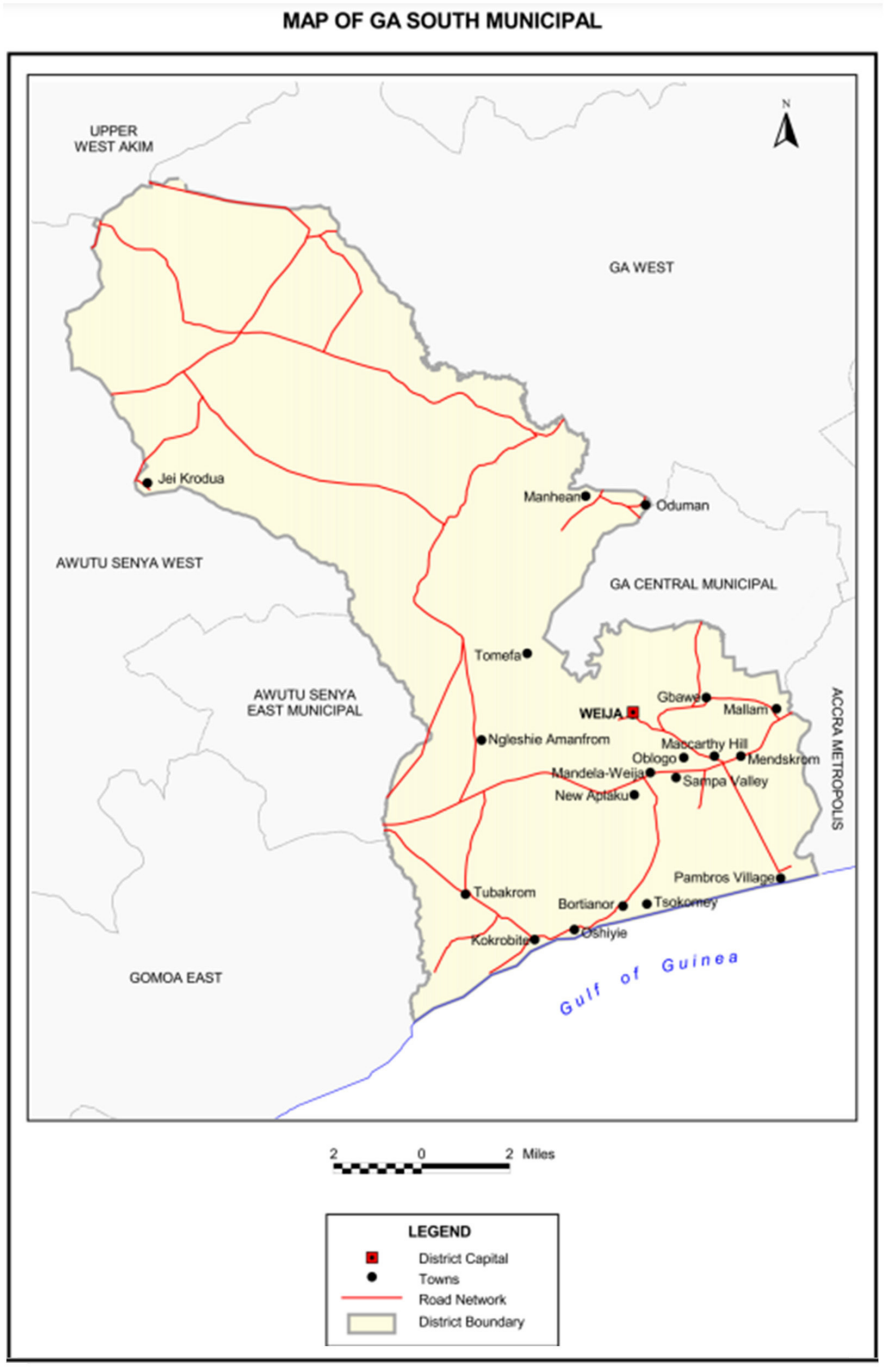
Map of Ga-South municipality. Reprinted with permission from Ghana Statistical Service (35).

The Ga-South municipality of the Greater Accra region of Ghana was purposively selected due to its mixed rural-urban characteristics and ease of access to the research team. The municipality is one of the two hundred and sixty-one (261) districts in Ghana. The Ga-South was carved out from the Ga West District in November 2007 and was established by Legislative Instrument 2,134 in July 2012 (35). It occupies a total land area of about 341.838 square kilometers with about 95 settlements. The municipality is made up of 20 sub-districts (Figure 1), with Ngleshie Amanfro being the largest sub-district and its district capital (35). The municipality currently has four (4) sub-municipals for health administration purposes; Ngleshie Amanfro, Bortianor, Kokrobite and Obom Sub Municipal (36). The Ngleshie Amanfrom Polyclinic is a high-level health facility in the Ngleshie Amanfro district, and the participants interviewed sought care including mental care at the facility.

### 2.3 Study participants

This study involved 17 participants: patients living with mental conditions, primary caregivers, mental health advocates, and health workers such as community psychiatry nurses and a health promotion officer. One interview was conducted with two participants, which accounts for the total number of interviews as 16 and the number of individual participants as 17. Table 1 provides a breakdown of participants.

**Table 1:** Number of participants.

**Participants**	**Number**
Patients living with mental illness	5
Primary caregivers	5
Mental health advocates	4
Health promotion	1
Psychiatry Nurses	2
**Total**	**17**

### 2.4 Recruitment and sampling

Participants were selected through purposive and snowball sampling methods at Ngleshie Amanfrom Polyclinic based on specific criteria relevant to the research. These criteria included participants being (a) mental health patients, and primary caregivers or relatives of patients with mental health conditions and who accessed any mental health service at the Ngleshie Amanfrom polyclinic and (b) further stakeholders such as healthcare providers, policymakers, advocates, and community leaders who shape or influence access to mental health care. The initial pool of individuals referred the research team to other participants who met the eligibility criteria and could potentially contribute and partake in the study. The exclusion criteria were individuals receiving mental health care primarily for substance abuse disorders, caregivers or relatives who are not directly involved in the care of the mental health patient, and temporary visitors of the mental health services at the polyclinic.

### 2.5 Data collection

Data collection was conducted by the qualitative research officers (ABA, LLY & NEEA) and research interns (RNBA, KANAA, NYA, EPKB, AA) of the STOP-NCD project over a period of 3 months, with a subsequent mop-up of data for 2 weeks. Primary data were collected via semi-structured in-depth interviews, each lasting ~5–60 min. Interviews were guided by a question guide which contained questions exploring participants' experiences in the provision and utilization of mental health services. The interview questions were generally set to explore and deepen our understanding of mental health conditions in country-specific contexts and to help contextualize available evidence of effective interventions. The guide explored the experiences, ideas, support, perceptions, power, enablers, and barriers to available mental health services and interventions in both formal and informal health settings. Supervision of the project was by IAA and TM.

### 2.6 Data analysis

All interviews were transcribed by six researchers (ABA, RNBA, KANAA, NYA, EPKB, and AA) with the local languages translated into English. This team reviewed the transcripts by cross-checking the transcripts against the audio recordings to reduce errors. ABA employed a thematic analysis approach to identify recurring themes and sub-themes within the data. A 6-phase coding framework for thematic analysis was used to identify themes and patterns in the data based on Clarke and Braun (8), which comprised: i) Familiarization of data, ii) Generation of codes, iii) Combining codes into themes, iv) Reviewing themes, v) Determine significance of themes, and vi) Reporting of findings. The analysis was conducted with NVivo 14 software.

### 2.7 Ethics

Ethical clearance was obtained from the Ghana Health Service Ethics Review Committee (protocol approval ID: GHS-ERC: 013/02/23) and the London School of Hygiene and Tropical Medicine (reference 28697). Written informed consent was obtained from all participants before any primary data collection. Data acquired were kept confidential, and the identities of participants were anonymized in reporting the results.

## 3 Results

The background characteristics of 17 participants are summarized in Table 2. Just over half were female and about a quarter were aged 31–40 followed by older age groups, about 40% had secondary school education, over half of participants were married with about a third being single, about 30% of participants had a monthly income of 1,000–1,500 Cedis (about USD 96). Amongst other stakeholders, two-thirds of health service providers had 6–10 years of experience and about half of participants had 1–5 years of experience with mental health advocacy with a quarter each having 6–10 and 16–20 years of advocacy experience.

**Table 2:** Demographic of participants.

**Demographic**	**Variables**	**Frequency (*N*)**	**%**
**Sex**	Male	8/17	47
	Female	9/17	53
**Age**	18–30	1/17	6
	31–40	4/17	24
	41–50	3/17	18
	51–60	3/17	18
	60+	2/17	12
**Level of education**	Primary/Middle school	2/17	12
	Junior High School	2/17	12
	Senior Secondary School/Technical School	7/17	41
	Tertiary	6/17	35
**Marital status**	Married	9/17	53
	Single	5/17	29
	Divorced	1/17	6
	Widowed	1/17	6
**Religious affiliation**	Christianity	15/17	88
	Islamic	1/17	6
**Ethnicity**	Akan	9/17	53
	Ga-adangme	3/17	18
	Ewe	3/17	18
	Gonja	1/17	6
**Monthly income of patients and caretakers (Ghana Cedis)**	< 500	2/10	15
	500–1,000	2/10	15
	1,000–1,500	3/10	30
**Years of professional experience as health workers**	1–5	1/3	33
	6–10	2/3	67
**Years of mental health advocacy**	1–5	2/4	50
	6–10	1/4	25
	11–15	-	-
	16–20	1/4	25

### 3.1 Thematic results

The reporting of findings is structured by the disparities identified at the community and facility levels. These findings were also thematized mainly under care management, stigmatization, and care accessibility under the two levels (Table 3).

**Table 3:** Thematic findings.

**Levels of care**	**Main themes**	**Sub-themes**
**Community level**	Care management	Low/No support from community and primary caregivers
		Inadequate community health nurses
		Inadequate mental health awareness campaigns
	Stigmatization	Stigma of patients
		Stigma of primary caregivers/relatives of patients
		Maltreatment of patients by traditional health practitioners
	Care accessibility	Inadequate funds support to seek care
		Inadequate care centers
**Facility level**	Care management	Inadequate diagnosis of cases
		Subpar application of mental health training by other healthworkers
		Delay in supply of medication
		Inadequate mental health staff
	Stigmatization	Stigmatization of mental health staff
		Isolation and neglect of patients in health facility
	Care accessibility	High cost of mental care services
		Inadequate supply of mental health drugs
		Referral and follow-up challenge

### 3.2 Community level findings

#### 3.2.1 Care management

At the community level, we found a notable deficiency in the support provided to individuals with mental health issues by both the community and primary caregivers. Furthermore, mental health awareness campaigns were reported to be inadequate, leaving many community members uninformed about mental health issues and the resources available to them. There was little or no support from the community leaders on mental health issues. Even though there were a lot of interventions targeting other non-communicable diseases in the community, the case was different for mental health. Mental health awareness campaigns to educate and inform the public were insufficient. Some participants mentioned that they had never seen or heard of any programme on mental health in the community, but there were many completed and ongoing programmes on HIV, Tuberculosis, diabetes, hypertension, and other health conditions. Some participants believed that there was not much priority on mental health compared to other conditions because mental health symptoms are not physical or can be seen on the body, unlike other conditions. Mental health patients lacked support from their relatives and felt neglected after diagnosis of their conditions which resulted in patients having to handle all treatment costs by themselves, as one participant stated:

“*I have been neglected by family members because of this condition and it has affected me financially.”* (Patient with Depression, male)

This lack of support by the community and relatives is compounded by the insufficient number of community health nurses available to address mental health needs. Some of the participants stated that there are not many community health nurses in the communities. Some communities in our study area did not have any community health nurse in their Community-based Health Planning and Services (CHPS) zones and compounds. Most of the mental health patients had to travel out of their communities to higher healthcare facilities to seek care. Patients compared how easy it was to access care for other health conditions like hypertension, malaria, diabetes and respiratory infections in the community than for mental health. An advocate added that the gap in awareness and support significantly hampers the effective management of mental health care within the community.

#### 3.2.2 Stigmatization

Our study revealed that stigmatization is a pervasive issue affecting both patients with mental health conditions and their primary caregivers or relatives. Patients often face significant social stigma, which can lead to isolation and discrimination. It was reported that this stigma extends to their caregivers, who may also experience social ostracism and judgment. Some participants highlighted that primary caregivers can abandon patients to avoid stigma by association. They expressed that patients are also labeled with derogatory names or terms, which foster an inaccurate association of common mental health conditions with severe psychiatric disorders, such as schizophrenia. One mental health advocate reflected:

“*So many examples, “Bodamfo” in Akan language which means a mad person. In the Ga language, other terms are used to describe people with mental health conditions which is also stigmatizing. About 2 weeks ago, we were in Ho to have a community durbar, and they also mentioned some of these things in their local language. And even to the media, you do not say someone is mentally challenged, it's wrong to say that. So, the media becomes stereotypes for using such words to describe a mental health patient.”* (Mental health advocate, male)

Additionally, we found that traditional health practitioners sometimes maltreat patients with mental health issues, further exacerbating the stigma and mistreatment these individuals face. Patients can be subjected to inhumane practices such as chaining and starvation as part of treatment in traditional care centers. A psychiatric nurse recounted:

“*Because of the belief system in our country and Africa in general, people would want to go to traditional healers and other faith-based healers for treatment. Because of the beliefs perceived that mental health patients are violent; the patients are chained and some of them are made to fast in terms of water and food.”* (Mental health advocate, male)

“*I have visited some prayer camps in the past. Sometimes, a patient is tied to a pole and remains there whether rain or shine, or they are made to fast for prolonged periods.”* (Psychiatric nurse, female)

This stigmatization creates a hostile environment that discourages individuals from seeking the care they need.

#### 3.2.3 Care accessibility

Participants reported that accessibility to mental health care is severely limited by financial constraints and the scarcity of care centers. Many individuals lack the necessary funds to seek appropriate mental health care, which is a significant barrier to treatment. Moreover, the number of care centers available is insufficient to meet the demand, leaving many without access to essential services. This lack of accessibility prevents timely and effective intervention, worsening the overall mental health outcomes in the community. A participant remarked:

“*I do not have money to go to the hospital that is why I am mostly home. There is no support from anyone or anywhere so there is nothing I can do about it.”* (Patient with Depression, male)

### 3.3 Facility level findings

#### 3.3.1 Care management

Within health care facilities, it was reported in our study that the management of mental health care is often inadequate. There are frequent failures in the proper diagnosis of mental health cases, which can lead to inappropriate or delayed treatment. Participants described instances in which general health staff would prematurely refer patients to psychiatric services without conducting thorough assessments:

“*The general nurses on the ward, at least they were taught some psychiatry, but let a patient misbehave a little, they will push the patient here. At least, do some tests, and run the labs—it might be an infection. In most cases, it is not a psychiatric condition.”* (Psychiatric nurse, female)

Another nurse echoed the sentiment:

“*I believe the doctors should assess them because sometimes it is not a psychiatric condition, and they just ask the patient to come. It is not only mental conditions that make someone hyperactive.”* (Psychiatric nurse, female)

Our analyses showed that most health facilities provide mental health training for all health professionals not only mental health clinicians, however, the application of mental health training among health care providers is sometimes inadequate, resulting in a lack of effective care practices. The health providers mentioned that the regularity of training was inadequate and the duration unsatisfactory. They also exclaimed that despite the training provided to health professionals, including nurses, there is an apparent disconnect between the training received and its practical application in mental health settings. One participant remarked:

“*Some of the midwives in the wards when talking to their clients, do not talk about psychiatric issues. Sometimes when there is an issue, they behave like they have not had any psychiatric training at all.”* (Psychiatry nurse, Female, 31years)

Additionally, some participants expressed that there are delays in the supply of necessary medications, which can disrupt treatment continuity. This was compounded by delays in receiving essential medical supplies for patients. One nurse expressed frustration over the pharmacy's failure to process medication requests:

“*With the pharmacy, we have been requesting for medications. When my in-charge asked some time back, she was told the request had been misplaced.”* (Psychiatry nurse, Female)

They exclaimed that the shortage of mental health staff further exacerbates these issues, as there are not enough trained professionals to meet the needs of patients. A psychiatric nurse added by mentioning that there are a few mental health staff in the facility, but most facilities in the district do not have mental health staff or psychiatrists at all.

#### 3.3.2 Stigmatization

Stigmatization within health care facilities affects both the staff and the patients. Mental health staff often face stigma, which can impact their professional and personal lives. Mental health workers reported experiencing stigmatization within their healthcare facilities, with colleagues often dismissing their expertise and contributions. A health promotion officer illustrated the casual disregard for mental health staff:

“*Even among the staff, we sometimes do things or say things that stigmatize the mental health staff. Sometimes a staff from that unit will be saying something, and you will just look at your friend and smile and say that what he or she is saying makes no sense. But if it is from any other person who is not from that unit, you will listen even if it does not make sense, you will still listen. But because the person is from the mental unit, when he speaks, you will say that what that person is saying does not make any sense.”* (Health promotion officer, Female)

Patients within these facilities are frequently isolated and neglected, which can lead to feelings of abandonment and exacerbate their mental health conditions. This environment of stigma and neglect undermines the quality of care provided and can deter individuals from seeking help. One advocate argued that psychiatric hospitals, by isolating patients, contribute to societal rejection rather than reintegration:

“*We feel that the isolation rather goes to dampening their state of mind. Rather, we must allow them to mingle with people they think they are of levels with.”* (Mental health advocate, male)

#### 3.3.3 Care accessibility

Our study revealed that the high cost of mental health services is a significant barrier to care for many individuals. Participants expressed that even when services are available, the financial burden can be prohibitive. Healthcare workers sometimes had to purchase drugs with their personal funds for patients. A participant described a patient's relapse following an inability to afford treatment:

“*We have a client who has really relapsed. When we mentioned the need for referral, the mother said she does not have that money. The patient has destroyed things and once attempted to stab an elderly woman. One facility mentioned 2,500 gh per month. Her money for a whole month will not even be up to the amount.”* (Psychiatric nurse, female)

Additionally, it was expressed that there is an inadequate supply of mental health drugs, which can lead to interruptions in treatment. Patients and primary caregivers expressed frustrations over medication shortages:

“*We need support with their drugs. It is difficult to get it. The last time we came here it was finished. One drugstore I went to, the drug has expired because people do not come for it since the government distributes it. I had to roam before I was able to get some.”* (Primary caregiver of mental health patient with Depression, Female)

Participants also revealed that the referral process and follow-up care also present challenges, as there are often delays and inefficiencies that hinder effective care continuity. Mental health nurses perform home visits to patients in the community, yet they lack transportation support, further straining the accessibility of care. These accessibility issues inhibit mental health care affordability and availability.

## 4 Discussion

Mental health care is shaped by several determinants at the individual, community, programmatic, system, and policy levels (2). These determinants either enable or hinder individuals from accessing mental health services (9). Barriers to mental health care can be sourced from inequitable treatment and may include concerns about the professionalism of providers and the inadequacy of care provided to individuals with mental illness (9). In both our community and facility findings, care accessibility was highly influenced by the availability of resources. Merati et al. (10) highlighted in their study that socioeconomic status plays a crucial role in mental health outcomes, with individuals from lower socioeconomic backgrounds facing more barriers to accessing care in comparison to individuals from higher socioeconomic backgrounds. The narrative is slightly different about urban-rural environments as recent studies showed that both equally played significant roles in shaping health outcomes (11).

Mental health disparities manifest globally and are reflected in the unequal treatment of patients and a lack of diversity in clinical trials (12). Contributing factors to these disparities include cultural perceptions, discrimination, stigma, limited awareness, and structural barriers to care based on religion, gender, and profession (13). This study reported that disparities in mental care occur in both the community and facility pertaining to care management, accessibility and stigmatization. Pitter and Khoury (14) report on how underdiagnosing mental health patients when they seek psychiatry services further exacerbates disparities (14). These findings align with global reports of systemic barriers in mental healthcare, particularly in low-resource settings, as described by Cook et al. (9). Disparities lead to poorer mental health outcomes, including worsening conditions due to substandard care (15). In many African contexts, such as Ghana, mental health remains a low priority, leading to poor management and neglect of critical mental health services (15). The consequences include increased risks of homelessness, suicidal behavior, job instability, and inadequate access to treatment. Additionally, untreated mental illness can lead to more severe conditions and even premature mortality from associated chronic diseases (9, 16, 17).

This study found that stigma is pervasive and affects both the patients and their caregivers and healthcare staff. This is similar to what Daliri et al. (18) reported in their study that caregivers of individuals with mental illnesses face significant challenges, including social stigma, financial strain, and emotional stress. Caregivers also experience physical symptoms like weight loss, and psychological effects such as anxiety, depression, and poor concentration, which compromise their ability to provide adequate care to patients (19, 20). Mental health patients are extremely concerned about disclosing their mental health status to their social networks and mental health professionals because of fear of their networks being stigmatized (9). Our findings revealed that health care workers are stigmatized just by their disposition to provide care to mental health patients. This is similar to what Cooper (19) reported that healthcare workers face moral distress and decreased job satisfaction due to systemic disparities in mental health care. The stigma surrounding mental health nursing reduces respect for the field, further complicating recruitment into the mental health workforce and negatively impacting the quality and provision of care to mental health patients (21). We found that all healthcare staff at the PHC level received training in mental healthcare. However, the application of skills has not been consistent perhaps due to lack of supportive supervision, unavailability of medicines and stigma. This echoes the literature highlighting the importance of a whole system approach to ensuring the quality of care (22, 23).

In sub-Saharan Africa, financial disparities hinder access to essential mental health services. On average, African governments allocate < $0.50 per capita to mental health, far below the World Health Organization's recommended $2 for low-income countries (24, 25). Our findings showed that inadequate financial resources and support is a major challenge in mental health services. In Ghana, < 1% of the national health budget is dedicated by the government to mental health (26). This funding is disproportionately focused on inpatient care, consequently incapacitating outpatient services to be severely underfunded (27). Other factors affecting mental health care in Ghana include fragmented health systems, weak referral pathways, and a shortage of trained mental health professionals, especially in rural areas (28). The absence of resources including poor quality of services and individual social barriers are also inhibitors of mental health care (29). A study by Ursula et al. (30) addressed the scarcity of trained mental health clinicians and epidemiological data on mental health. They reported that these factors hinder effective mental health interventions, creating significant challenges in accessibility and quality of care in Ghana (30).

Addressing mental health disparities will require a multifaceted approach, including policy reforms, community engagement, and integrating mental health services into primary health care (24, 31, 34). Community-based Health Planning and Services (CHPS) which was classified in a study by Adusei et al. (32) as Ghana's primary strategy for providing close-to-client healthcare delivery should spearhead the integration of mental services in their deliverables. Efforts should also focus on improving access to care, addressing health beliefs, and reducing stigma within racial and minority groups (9). Prioritizing mental health in national health policies and fostering collaboration among stakeholders is essential to building a more equitable system that addresses mental health disparities.

### 4.1 Strength, limitations, and future direction of study

The strength of this study is its use of in-depth interviews with diverse stakeholders, providing rich qualitative data on mental health disparities. The inclusion of both patients and healthcare workers offers a comprehensive view of the challenges faced. However, the findings are limited by the small sample size and the focus on one district in Ghana, which may not be representative of other districts in the country. The study employed purposive and snowball sampling which have several limitations, including selection bias and the sample being less representative of the broader population and consequently limited generalizability of the findings. There was also a risk of social desirability bias, where participants may give responses influenced by social relationships rather than their true experiences. The future direction of this study should be geared toward including more districts in Ghana and employing a different probability sampling method.

## 5 Conclusion

This study reveals critical disparities in access to mental health care in Ga-South, Ghana. The stigma surrounding mental health affects patients, caregivers, and healthcare providers hinders effective care delivery. The challenges include limited availability of trained mental health professionals, inflated costs of treatment and medication, and inadequate mental health awareness, which contribute to delayed healthcare-seeking behavior. The findings underscore the need for interventions that prioritize mental health at the primary care level, enhance healthcare worker training, and address stigma through community-based initiatives. Additionally, improving mental health resource allocation in low-resource settings like Ghana is essential for equitable and effective healthcare access.

## Data Availability

The raw data supporting the conclusions of this article will be made available by the authors, without undue reservation.
